# Supplementary material for: Graphene mechanical pixels for Interferometric Modulator Displays
Source: Nat Commun. 2018 Nov 16;9:4837. doi: 10.1038/s41467-018-07230-w (PMC6240083; doi:10.1038/s41467-018-07230-w)
Supplement: Supplementary file 1 — Supplementary Information [file 41467_2018_7230_MOESM1_ESM.pdf]

Supplementary Information for: Graphene mechanical pixels for Interferometric Modulator Displays

Santiago J. Cartamil-Bueno[1,*], Dejan Davidovikj[2], Alba Centeno[3], Amaia Zurutuza[3], Herre S.J. van der Zant[2], Peter G. Steeneken[2] & Samer Houri[2,*‡]

[1]*SCALE Nanotech OÜ, Sepapaja 6, Tallin 15551, Estonia*

[2]*Kavli Institute of Nanoscience, Delft University of Technology, Lorentzweg 1, 2628CJ, Delft, The Netherlands*

[3]*Graphenea SA, 20018 Donostia-San Sebastián, Spain*

[*]*Corresponding authors: cartamil@scalenano.tech, Houri.Samer@lab.ntt.co.jp.*

Keywords: CVD graphene, electro-optic modulators, MEMS, display, GIMOD

[‡]Current affiliation: NTT Basic Research Laboratories, NTT Corporation, 3-1 Morinosato-Wakamiya, Atsugi, Kanagawa 243-0198, Japan

Supplementary Figures

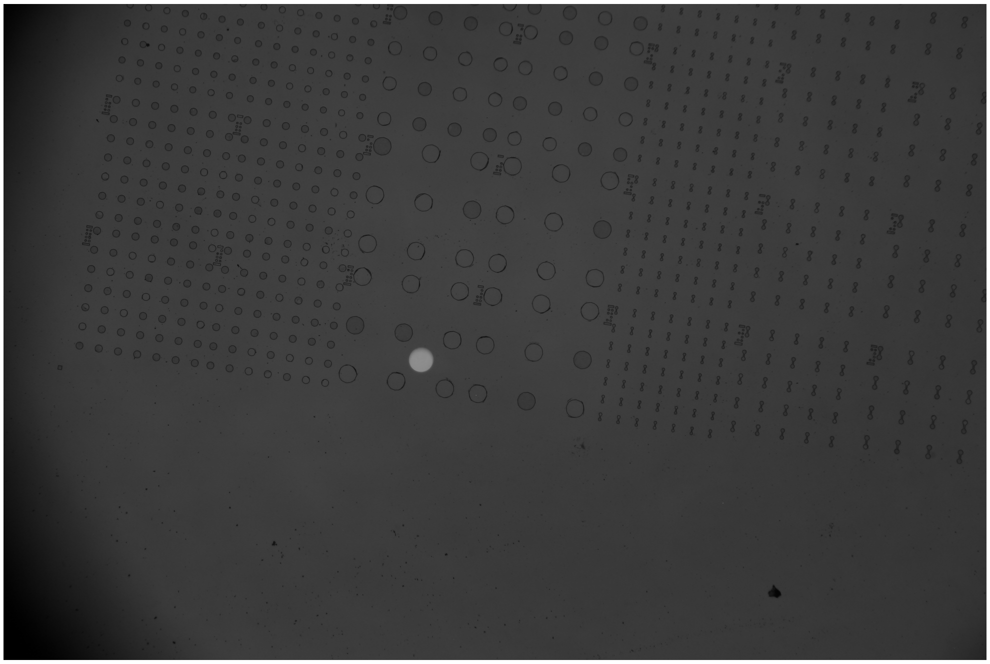

Supplementary Figure 1: Optical microscope image showing the light collection area of the spectrometer. Here the spectrometer was replaced by a light source to project a spot on the sample.

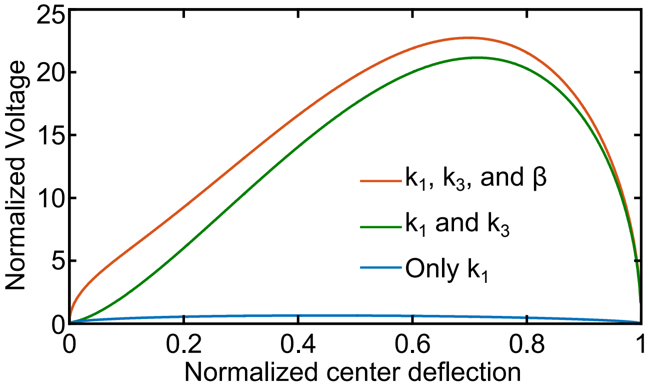

Supplementary Figure 2: The voltage-deflection curves for pure linear stiffness (blue), combined linear and cubic stiffness (green), and combined linear, cubic and hydrostatic effects (red), as obtained for $k_3 = 1000k_1$, and $\beta = 100k_1$.

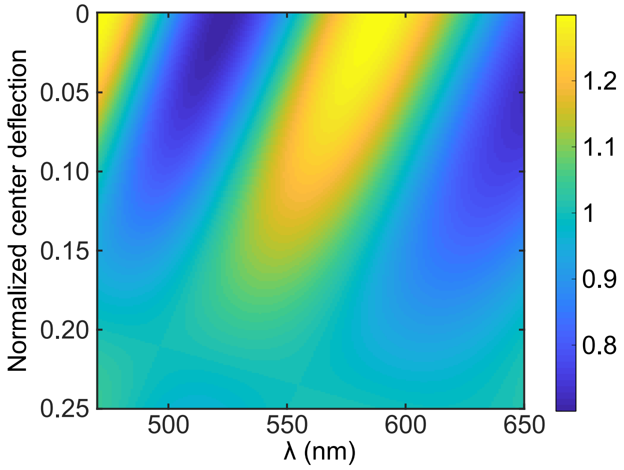

Supplementary Figure 3: Simulation of the normalized drum averaged reflectance based on Supplementary Equation 10, using values for $A(\lambda)$ and $B(\lambda)$ from the fitted data.

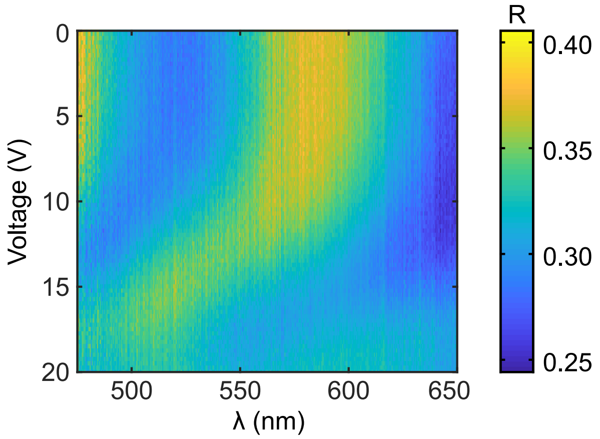

Supplementary Figure 4: Measured drum averaged reflection as obtained from spectrometer data, shown as a function of electrostatic actuation voltage.

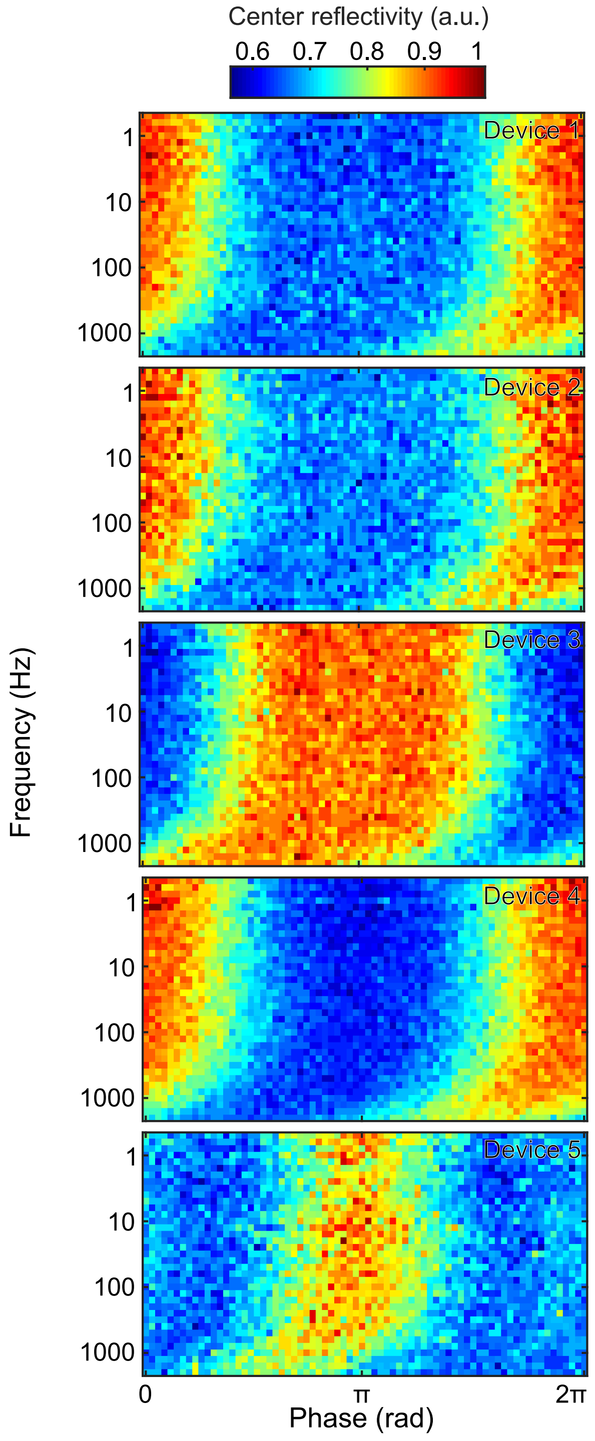

Supplementary Figure 5: Experimentally obtained normalized reflectance at the center of all drums as a function of frequency and phase for all the drums measured stroboscopically. The same phase delay (from the signal amplifier) is observed as in Figure 3b from the main text.

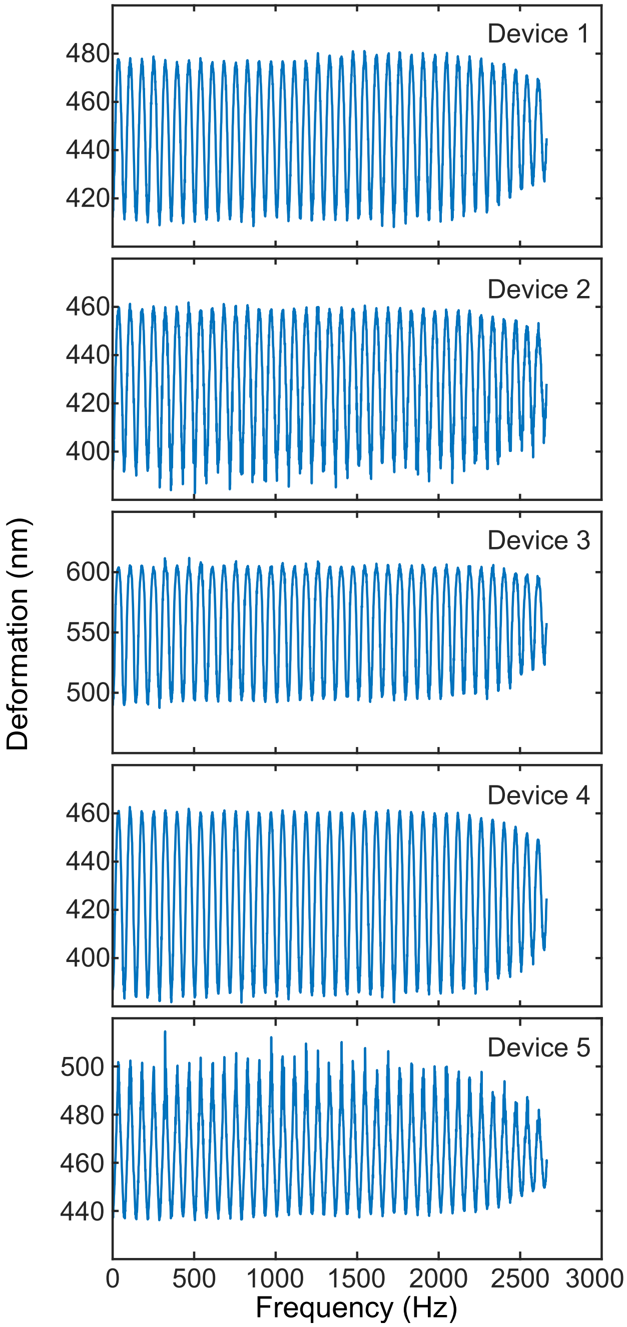

Supplementary Figure 6: Displacement as extracted from stroboscopic measurement shown as a function of frequency for all devices. Note the larger response seen in Device 3.

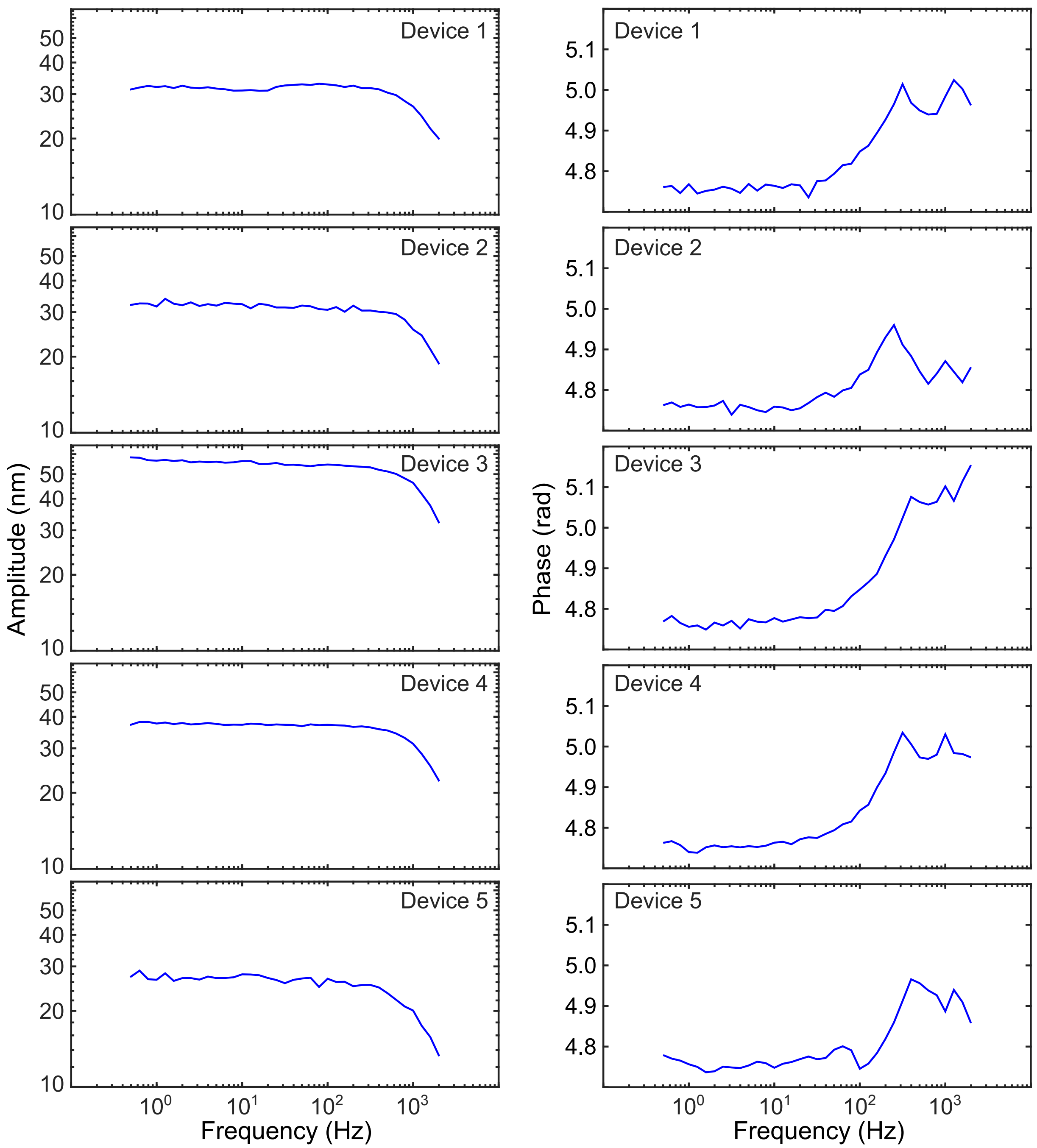

Supplementary Figure 7: Phase and amplitude Bode plots for all strobscopically measured devices, showing that the response is flat up to 400 Hz (cutoff frequency of amplifier).

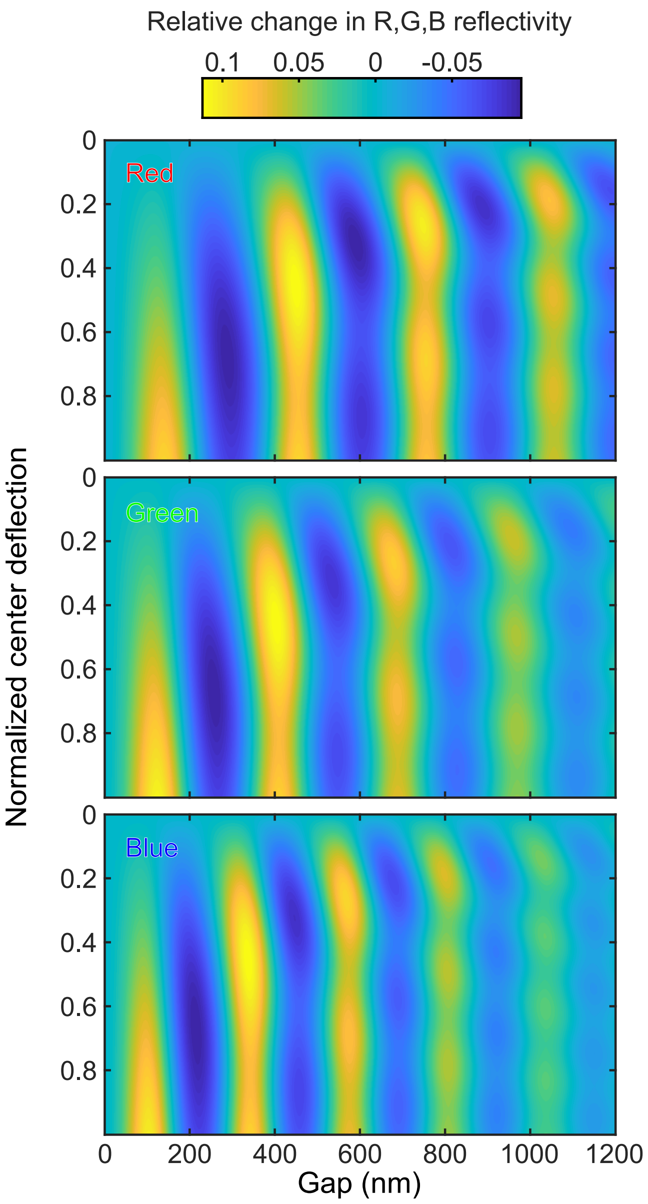

Supplementary Figure 8: Simulated relative change in the R, G, B indices for a GIMOD pixel of 5 μm in diameter, under halogen lamp illumination.

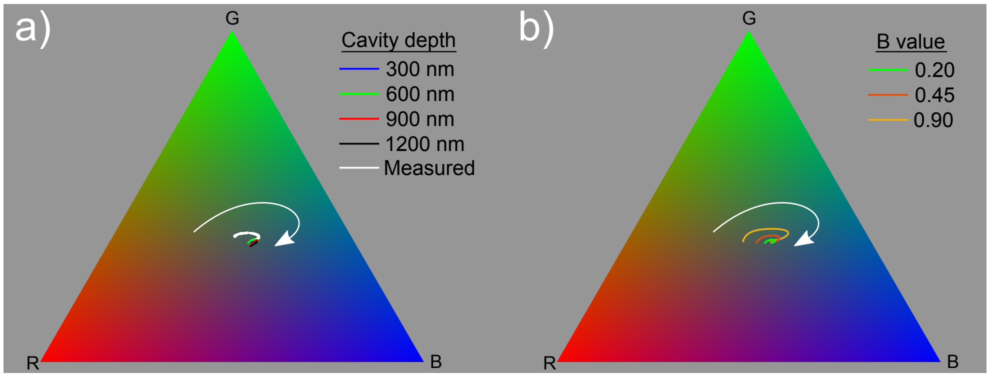

Supplementary Figure 9: Simulated gamut pixel trajectory on a sRGB color triangle (CIE1931 colorimetry). a) Simulation of the average reflectance of a GIMOD pixel (5 μm in diameter) for different depths of the cavity (A=1, B=0.2). b) Simulation of the pixel trajectory for cavity depth of 600 nm, halogen illumination, and different values of B (A=1).

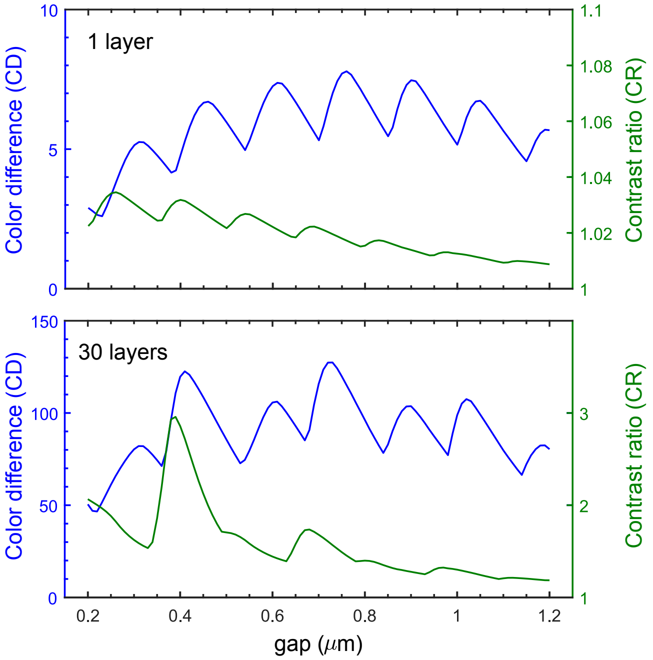

Supplementary Figure 10: Simulated color difference (CD) and contrast ratio (CR) of a GIMOD pixel for 1 layer (top panel) and 30 layers (bottom panel).

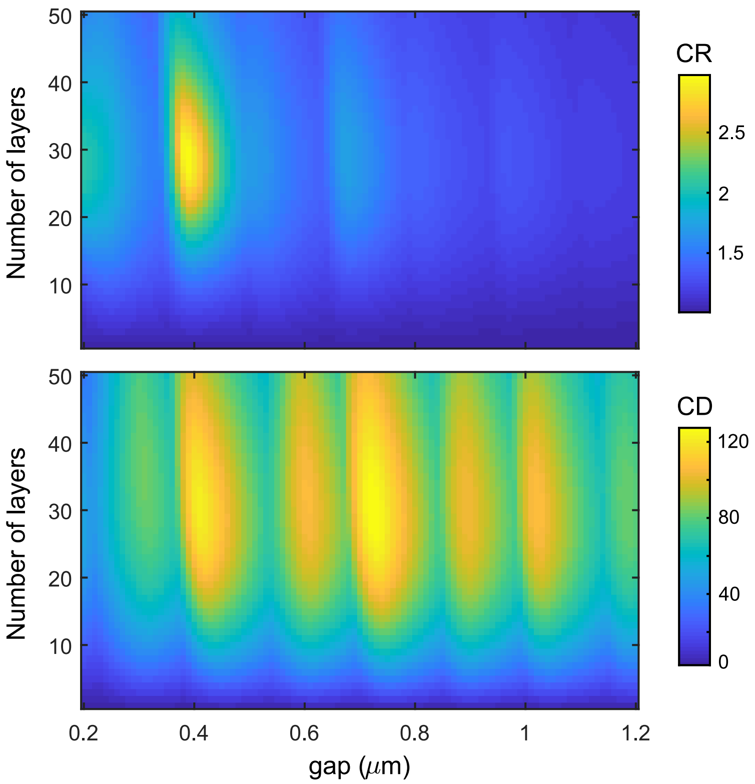

Supplementary Figure 11: Simulated contrast ratio (CR, top panel) and color difference (CD, bottom panel) for a GIMOD pixel with a number of layers ranging from 1 to 50 layers.

Supplementary Note 1: Area measured by spectrometer

For the spectrometer measurements, we used a focused fiber to collect the reflected light from the graphene drumheads. In order to align the fiber so it points and covers the desired device, we first connect the fiber to a light source so we can observe a bright spot on top of the sample. Supplementary Figure 1 shows the light spot of 27.5 μm in diameter located away from the devices, covering an area of graphene on SiO_2.

Supplementary Note 2: Capacitance of the deflected circular membrane

We apply a 1 degree of freedom graphene membrane model that neglects bending rigidity, and describes the drums' deflection by an axisymmetric parabolic profile given by $\delta(r) = \delta_c \left(1 - (\frac{r}{a})^2\right)$, where δ_c is the center deflection, a is the radius of the drum, and r is the radial distance away from the drum's center. The capacitance of the circular membrane is now given by:

$$C = \int_0^{2\pi} \int_0^a \frac{\epsilon_0 r dr d\theta}{g_0 - \delta(r)} \qquad \text{(Supplementary Equation 1)}$$

We define $\bar{\delta} = \frac{\delta_c}{g_0}$, $Y = \left(1 - \frac{r^2}{a^2}\right)$, and $C_0 = \frac{\epsilon_0 \pi a^2}{g_0}$, and re-express Supplementary Equation 1 as:

$$\begin{aligned} C = -C_0 \int_1^0 \frac{dY}{1 - \bar{\delta}Y} = \frac{C_0}{\bar{\delta}} \int_1^0 \frac{d(1 - \bar{\delta}Y)}{1 - \bar{\delta}Y} \\ C = -C_0 \frac{ln(1 - \bar{\delta})}{\bar{\delta}} \end{aligned} \qquad \text{(Supplementary Equation 2)}$$

Supplementary Note 3: Electrostatic force on a circular graphene membrane

We obtain the electrostatic force under the effect of an applied voltage V by taking the spatial derivative of the electrostatic potential energy, thus:

$$\begin{aligned} F_{Elec}(\delta_c, V) = -\frac{d}{d\delta_c}\frac{1}{2}CV^2 = -\frac{1}{g_0}\frac{d}{d\bar{\delta}}\frac{1}{2}CV^2 \\ F_{Elec} = -\frac{C_0 V^2}{2g_0}\left(\frac{\bar{\delta} - (\bar{\delta} - 1)ln(1 - \bar{\delta})}{(\bar{\delta} - 1)\bar{\delta}^2}\right) \end{aligned} \qquad \text{(Supplementary Equation 3)}$$

Supplementary Note 4: Force equilibrium equation

The deflection of the graphene membrane, if the perfect hermeticity assumption is to be maintained, requires that the force balance equation accounts for the hydrostatic pressure that develops from the compression of the gas trapped within the cavity and cannot escape it. To calculate this

last effect, we assume that the parabolic profile of membrane deflection applies and that the compression is isothermal, thus applying the perfect gas law the gas pressure within the cavity reads:

$$P = \frac{Ag_0 P_0}{V} = \frac{Ag_0 P_0}{A(g_0 - \frac{\delta_c}{2})} = \frac{P_0}{1 - \frac{\bar{\delta}}{2}}, \qquad \text{(Supplementary Equation 4)}$$

where A is the area of the circular membrane, P_0 is the initial (ambient) pressure, and the factor $\frac{1}{2}$ in the denominator on the rightmost hand side term comes from integrating the area under the parabolic profile [1]. Thus the hydrostatic force acting on the membrane due to the change in the cavity pressure can be written as:

$$F_{Hydro} = A\Delta P = A(P - P_0) = AP_0 \frac{\bar{\delta}/2}{1 - \bar{\delta}/2} \qquad \text{(Supplementary Equation 5)}$$

Note that Supplementary Equation 5 applies in case of both positive and negative deflection, i.e. positive and negative differential pressure.

Combining all the terms we obtain the following force equilibrium equation:

$$k_1\delta_c + k_3\delta_c^3 + F_{Hydro} = F_{Elec}$$

$$=> k_1 g_0 \bar{\delta} + k_3 g_0^3 \bar{\delta}^3 + AP_0 \frac{\bar{\delta}/2}{1 - \bar{\delta}/2} = -\frac{C_0 V^2}{2g_0}\left(\frac{\bar{\delta} - (\bar{\delta} - 1)ln(1 - \bar{\delta})}{(\bar{\delta} - 1)\bar{\delta}^2}\right)$$

(Supplementary Equation 6)

Supplementary Equation 6 can be rewritten in the following non-dimensional form:

$$\bar{\delta} + \bar{k_3}\bar{\delta}^3 + \beta \frac{\bar{\delta}/2}{1 - \bar{\delta}/2} = -\frac{\bar{V}^2}{2}\left(\frac{\bar{\delta} - (\bar{\delta} - 1)ln(1 - \bar{\delta})}{(\bar{\delta} - 1)\bar{\delta}^2}\right), \qquad \text{(Supplementary Equation 7)}$$

where the non-dimensional parameters are given as $\bar{k_3} = k_3 g_0^2/k_1$, $\beta = AP_0/k_1 g_0$, and $\bar{V}^2 = C_0 V^2/k_1 g_0$.

Typical values of $\bar{k_3}$ range between $10^{-1}-10^{4}$, of β between $1-100$, and $\bar{V}^2$ between $(10^{-3}-1)V^2$. In Supplementary Figure 2 the voltage displacement curve is plotted according to Supplementary Equation 7 to show the impact of nonlinear stiffness and gas compression on the mechanical susceptibility of the device.

Supplementary Note 5: Optical response of deflected drums

The optical reflectivity model used neglects any cavity effects and treats the suspended drum as an absorbing layer placed in front of the silicon back mirror. This simplification is valid for thin graphene membranes, less than 5 layers [2], and leads to the drum reflectance

$$R(r,\lambda) = A(\lambda) + B(\lambda)\cos(\phi - \pi\bar{X}(r)), \qquad \text{for } \bar{X}(r), \qquad \text{(Supplementary Equation 8)}$$

where λ is the wavelength, $A(\lambda)$ and $B(\lambda)$ are wavelength dependent constants, $\phi = \frac{4\pi g}{\lambda} + \phi'$ is a phase shift induced by the optical travel path and by the graphene, $\hat{X}\,(r) = \frac{4g\bar{\delta}(r)}{\lambda}$ and $\delta(r) = \delta_c\left(1 - (\frac{r}{a})^2\right)$. Taking nominal values for the optical properties of graphene, we can neglect ϕ' as it is negligibly small compared to the travel path term. By integrating the reflectance over the drum's area, we obtain:

$$R_{drum}\,(\lambda) = \int_0^{2\pi}\int_0^{a} R\,(r,\lambda)\, rdrd\theta$$

$$=> R_{drum}\,(\lambda) = 2\pi\left[\frac{-a^2}{2}\int_1^0 A(\lambda) + B(\lambda)cos\left(\phi - \frac{4\pi\delta_c}{\lambda}Y\right)dY\right]$$

(Supplementary Equation 9)

where $Y = \left(1 - \frac{r^2}{a^2}\right)$. Dividing Supplementary Equation 9 by πa^2 to obtain the drum's average reflectivity, and integrating we arrive at:

$$R_{avg}(\lambda) = A(\lambda) + B(\lambda)\frac{\lambda}{4\pi\delta_c}\left[sin(\phi) - sin\left(\phi - \pi\frac{4\delta_c}{\lambda}\right)\right] \quad \text{(Supplementary Equation 10)}$$

Supplementary Figure 3 shows the simulated spectral response, as obtained from Supplementary Equation 10, of a graphene drum as a function of center deflection for a gap of 1140 nm. Supplementary Figure 4 shows the experimentally obtained spectral response as a function of voltage for the same gap.

Supplementary Note 6: Stroboscopic measurement of graphene drums/pixels

Supplementary Figures 5, 6 and 7 present the results of stroboscopic characterization for the all the measured devices. The device shown in the main text corresponds to Device 4 in the figures.

Supplementary Note 7: Simulating the RGB response of GIMOD pixels

The RGB response of a GIMOD pixel can be obtained using the following equation:

$$Red, Green, Blue = \int_0^\infty I(\lambda) C_{Red,Green,Blue}(\lambda) R_{avg}(\lambda) d\lambda, \quad \text{(Supplementary Equation 11)}$$

where $R_{avg}(\lambda)$ is the wavelength dependent drum average reflectivity obtained in Supplementary Equation 10, $I(\lambda)$ is the power spectrum of the illumination source, and $C_{Red,Green,Blue}$ is the CIE 1931 color matching functions for Red, Green, and Blue respectively [3]. The left hand side simply indicates the value for the Red, Green, or Blue.

The values obtained from Supplementary Equation 11, still need to undergo a gamma compression

to account for the way human vision perceives colors. This is done by applying the following transformation:

$$RGB_{Corrected} = RGB^{\gamma} \qquad \text{(Supplementary Equation 12)}$$

In this work, the value for gamma in Supplementary Equation 12 is taken to be $\gamma = \frac{1}{2.4}$, while the illumination profile $I(\lambda)$ used in these simulations is that of a halogen lamp [4]. The Red, Green, and Blue components for a GIMOD pixel are obtained numerically for various gaps and for A, B values fitted experimentally (A=0.32, B=0.063), the relative change in each color component as a function of center deflection and gap size is shown in Supplementary Figure 8.

In order to plot these values as trajectories on an RGB color triangle, the following coordinate transformation is applied [3]:

$$x = \frac{Blue}{Red + Green + Blue}$$
$$y = \frac{Green}{Red + Green + Blue} \qquad \text{(Supplementary Equation 13)}$$

Various trajectories of these GIMOD pixels on an RGB color triangle are equally plotted in Supplementary Figure 9.

Supplementary Note 8: Optimizing the performance of GIMOD pixels

In order to find out what would be an optimal thickness, i.e. number of graphene layers, for a GIMOD pixel we resort to more detailed simulations. These simulations are based on a full optical model that accounts for the multiple reflectance and absorbance of the successive optical layers, i.e. air-graphene-air-silicon, as described in [5]. According to this optical model, the reflectance of a

graphene layer suspended on top of a Silicon cavity is given as:

$$
\begin{aligned}
R = & |2r_1 e^{i\phi_2}\sin(\phi_1) + r_2 e^{-(\phi_1+\phi_2)} \\
& - \frac{r_1^2 r_2 e^{i(\phi_1-\phi_2)}}{e^{i(\phi_1+\phi_2)} + r_1^2 e^{-i(\phi_1-\phi_2)} + 2r_1 r_2 e^{-i\phi_2}\sin(\phi_1)}|,
\end{aligned}
\qquad \text{(Supplementary Equation 14)}
$$

where r_1 and r_2 are the Fresnel reflection coefficients of air-graphene and air-silicon interfaces, respectively. ϕ_1 and ϕ_2 are the phase changes induced by the optical path through the graphene and the cavity respectively.

Next we maintain the assumed parabolic deflection profile of the graphene film, this is reasonable since literature suggests that 2D materials continue to act as membranes for thicknesses up to 50 layers [6]. In order to obtain the drum averaged reflection of the GIMOD, we perform the following integral:

$$R(\lambda) = \int_0^{2\pi} \int_0^a R(r,\lambda) r dr d\lambda. \qquad \text{(Supplementary Equation 15)}$$

Unlike in section 5, the function $R(r,\lambda)$ is now obtained from Supplementary Equation 14, and the integration is performed numerically. Thereafter the color response is obtained following the procedure detailed in section 7 (Supplementary Equations 11-13), here we use the X, Y, Z, color matching functions for convenience. As figure of merits to judge the GIMOD performance as a function of thickness, we use the color difference and the contrast ratio [7,8]. The contrast ratio (CR) between two points in color space is defined as follows.

$$CR = \frac{L_1^*}{L_2^*}, \qquad \text{(Supplementary Equation 16)}$$

where L^* is the "Lightness" obtained from the CIE standard from:

$$L^*(Y) = \begin{cases} \left(\frac{116}{12}\right)^3 Y, & \text{for } Y \leq \left(\frac{24}{116}\right)^3 \\ 116Y^{1/3} - 16, & \text{for } \left(\frac{24}{116}\right)^3 < Y \end{cases} \qquad \text{(Supplementary Equation 17)}$$

Whereas the color difference (ΔE) between two points in color space is defined as:

$$\Delta E = \sqrt{(L_2^* - L_1^*)^2 + (u_2^* - u_1^*)^2 + (v_2^* - v_1^*)^2}, \qquad \text{(Supplementary Equation 18)}$$

$$\text{with} \quad \begin{cases} u^* = 13L^*(u' - u_0') \\ v^* = 13L^*(v' - v_0') \end{cases} \quad \text{and} \quad \begin{cases} u' = \frac{4x}{3-2x+12y} \\ v' = \frac{9y}{3-2x+12y} \end{cases}$$

where x,y are the normalized tri-stimulus coordinates, and u_0' and v_0' are the u' and v' values for the illumination source. For the halogen illumination source assumed in this work $u_0' = 0.1946$, and $v_0' = 0.4711$. Note that the above definition correspond to color difference in the CIELUV standard. As an example, Supplementary Figure 10 shows the color difference and contrast ratio for a single layer graphene (top panel) and a 30 layers graphene GIMOD pixels (bottom panel).

We repeat the previous simulation for a range of graphene layers going from 1 to 50, the resulting contrast ratio and color difference obtained are shown in the two dimensional plots of Supplementary Figure 11. From these simulations we conclude that the number of graphene layers that provide the highest contrast ratio and richest color gamut is around 29 layers, with a corresponding optimal cavity gap of around 390 nm. Note that color difference values have a maximum value around gap of 740 nm, however that cavity depth corresponds to a low contrast ratio. A gap on the order of 400 nm thus offers the best values for both color difference and contrast ratio.

Supplementary References

1. Cartamil-Bueno, S. J., Steeneken, P. G., Centeno, A., Zurutuza, A., van der Zant, H. S. J. & Houri, S. Colorimetry Technique for Scalable Characterization of Suspended Graphene. *Nano Letters* **16**, 6792–6796 (2016).

2. Reserbat-Plantey, A., Schädler, K. G.,Gaudreau, L., Navickaite, G., Güttinger, J., Chang, D., Toninelli, C., Bachtold, A. & Koppens, F. H. *Electromechanical control of nitrogen-vacancy defect emission using graphene NEMS.*. *Nature Communications* **7** 10218 (2016).

3. Westland, S., Ripamonti, C. & Cheung, V. *Computational colour science using MATLAB*. *John Wiley & Sons* (2012).

4. `https://www.thorlabs.com/newgrouppage9.cfm?objectgroup_id=7541`

5. Blake, P. & Hill, E. Making graphene visible. *Applied Physics Letters* **063124**, 3 (2007).

6. Cartamil-Bueno, S. J., Steeneken, P. G., Tichelaar, F. D., Navarro-Moratalla, E., Venstra, W. J., van Leeuwen, R., Coronado, E., van der Zant, H. S. J., Steele, G. A. & Castellanos-Gomez, A. High-quality-factor tantalum oxide nanomechanical resonators by laser oxidation of $TaSe_2$. *Nano Research* **8**, 2842–2849 (2015).

7. Poynton, C. *Digital video and HD: Algorithms and Interfaces*. *Elsevier* (2012).

8. Ozawa, R., Mada, H., Shimomura, T., Mizoguchi, R. & Kobayashi, S. Colorimetric Characteristics of Neutral Polarizers. *Japanese Journal of Applied Physics* **19**, L453 (1980).
