# Supplementary material for: Graphene mechanical pixels for Interferometric Modulator Displays
Source: Nat Commun. 2018 Nov 16;9:4837. doi: 10.1038/s41467-018-07230-w (PMC6240083; doi:10.1038/s41467-018-07230-w)
Supplement: Supplementary file 3 — Description of Additional Supplementary Files [file 41467_2018_7230_MOESM3_ESM.pdf]

Description of Additional Supplementary Files

File Name: Supplementary Movie
Description: GIMOD prototype showing a static image (Graphene Flagship logo) whose 5µm-in-diameter pixels blink between yellow and blue.
